# Potentiation of anti-cancer drug activity at low intratumoral pH induced by the mitochondrial inhibitor *m*-iodobenzylguanidine (MIBG) and its analogue benzylguanidine (BG)

**DOI:** 10.1038/sj.bjc.6690127

**Published:** 1999-02

**Authors:** A Kuin, M Aalders, M Lamfers, D J van Zuidam, M Essers, J H Beijnen, L A Smets

**Affiliations:** Department of Experimental Therapy, The Netherlands Cancer Institute, Antoni van Leeuwenhoek Hospital, Amsterdam, The Netherlands; Department of Pharmacy and Pharmacology, The Netherlands Cancer Institute, Slotervaart Hospital, Amsterdam, The Netherlands

**Keywords:** tumour pH, mitochondrial inhibition, intracellular pH, anti-cancer drugs

## Abstract

Tumour-selective acidification is of potential interest for enhanced therapeutic gain of pH sensitive drugs. In this study, we investigated the feasibility of a tumour-selective reduction of the extracellular and intracellular pH and their effect on the tumour response of selected anti-cancer drugs. In an in vitro L1210 leukaemic cell model, we confirmed enhanced cytotoxicity of chlorambucil at low extracellular pH conditions. In contrast, the alkylating drugs melphalan and cisplatin, and bioreductive agents mitomycin C and its derivative EO9, required low intracellular pH conditions for enhanced activation. Furthermore, a strong and pH-independent synergism was observed between the pH-equilibrating drug nigericin and melphalan, of which the mechanism is unclear. In radiation-induced fibrosarcoma (RIF-1) tumour-bearing mice, the extracellular pH was reduced by the mitochondrial inhibitor *m*-iodobenzylguanidine (MIBG) or its analogue benzylguanidine (BG) plus glucose. To simultaneously reduce the intracellular pH, MIBG plus glucose were combined with the ionophore nigericin or the Na^+^/H^+^ exchanger inhibitor amiloride and the Na^+^-dependent HCO_3_^−^/Cl^−^exchanger inhibitor 4,4′-diisothiocyanostilbene-2,2′-disulphonic acid (DIDS). Biochemical studies confirmed an effective reduction of the extracellular pH to approximately 6.2, and anti-tumour responses to the interventions indicated a simultaneous reduction of the intracellular pH below 6.6 for at least 3 h. Combined reduction of extra- and intracellular tumour pH with melphalan increased the tumour regrowth time to 200% of the pretreatment volume from 5.7 ± 0.6 days for melphalan alone to 8.1 ± 0.7 days with pH manipulation (*P*< 0.05). Mitomycin C related tumour growth delay was enhanced by the combined interventions from 3.8 ± 0.5 to 5.2 ± 0.5 days (*P*< 0.05), but only in tumours of relatively large sizes. The interventions were non-toxic alone or in combination with the anti-cancer drugs and did not affect melphalan biodistribution. In conclusion, we have developed non-toxic interventions for sustained and selective reduction of extra- and intracellular tumour pH which potentiated the tumour responses to selected anti-cancer drugs. *1999 Cancer Research Campaign*


*British Journal of Cancer* (1999) **79**(5/6), 793–801
© 1999 Cancer Research Campaign
Article no. bjoc.1998.0127

### Potentiation of anti-cancer drug activity at low intratumoral pH induced by the mitochondrial inhibitor *m*-iodobenzylguanidine (MIBG) and its analogue benzylguanidine (BG)

**A Kuin[1], M Aalders[1], M Lamfers[1], DJ van Zuidam[1], M Essers[1], JH Beijnen[2] and LA Smets[1]**

[1]Department of Experimental Therapy, The Netherlands Cancer Institute, Antoni van Leeuwenhoek Hospital and [2]Department of Pharmacy and Pharmacology, Slotervaart Hospital, The Netherlands Cancer Institute, Amsterdam, The Netherlands

**Summary** Tumour-selective acidification is of potential interest for enhanced therapeutic gain of pH sensitive drugs. In this study, we investigated the feasibility of a tumour-selective reduction of the extracellular and intracellular pH and their effect on the tumour response of selected anti-cancer drugs. In an in vitro L1210 leukaemic cell model, we confirmed enhanced cytotoxicity of chlorambucil at low extracellular pH conditions. In contrast, the alkylating drugs melphalan and cisplatin, and bioreductive agents mitomycin C and its derivative EO9, required low intracellular pH conditions for enhanced activation. Furthermore, a strong and pH-independent synergism was observed between the pH-equilibrating drug nigericin and melphalan, of which the mechanism is unclear. In radiation-induced fibrosarcoma (RIF-1) tumour-bearing mice, the extracellular pH was reduced by the mitochondrial inhibitor *m*-iodobenzylguanidine (MIBG) or its analogue benzylguanidine (BG) plus glucose. To simultaneously reduce the intracellular pH, MIBG plus glucose were combined with the ionophore nigericin or the $Na^+/H^+$ exchanger inhibitor amiloride and the $Na^+$-dependent $HCO_3^-/Cl^-$ exchanger inhibitor 4,4′-diisothiocyanostilbene-2,2′-disulphonic acid (DIDS). Biochemical studies confirmed an effective reduction of the extracellular pH to approximately 6.2, and anti-tumour responses to the interventions indicated a simultaneous reduction of the intracellular pH below 6.6 for at least 3 h. Combined reduction of extra- and intracellular tumour pH with melphalan increased the tumour regrowth time to 200% of the pretreatment volume from 5.7 ± 0.6 days for melphalan alone to 8.1 ± 0.7 days with pH manipulation ($P < 0.05$). Mitomycin C related tumour growth delay was enhanced by the combined interventions from 3.8 ± 0.5 to 5.2 ± 0.5 days ($P < 0.05$), but only in tumours of relatively large sizes. The interventions were non-toxic alone or in combination with the anti-cancer drugs and did not affect melphalan biodistribution. In conclusion, we have developed non-toxic interventions for sustained and selective reduction of extra- and intracellular tumour pH which potentiated the tumour responses to selected anti-cancer drugs.

**Keywords:** tumour pH; mitochondrial inhibition; intracellular pH; anti-cancer drugs

In tissue culture models, low pH is a promising target for improved chemotherapy (Wike-Hooley et al, 1984; Volk et al, 1993). An acidic environment enhances the uptake of weak acids (Mikkelsen et al, 1985; Jähde et al, 1989) and topoisomerase I inhibitors (Gabr et al, 1997), it also increases the activation of bioreductive agents (Gibson et al, 1992; Philips et al, 1992; Begleiter and Leith, 1993) and potentiates the interaction of alkylating agents and platinum-containing drugs with DNA (Jähde et al, 1989; Atema et al, 1993). Because conditions of low pH in cell culture include a simultaneous reduction in the intracellular pH (Atema et al, 1993), studies in vitro cannot discriminate between the contribution of reduced extra- or intracellular pH in the observed potentiations.

Tumour-selective acidification can be achieved in animal and human tumours by stimulation of their intrinsically high glycolytic flux by glucose infusion alone (Jähde and Rajewsky, 1982; Thislethwaite et al, 1987) or with coadministration of the mitochondrial inhibitor *m*-iodobenzylguanidine (MIBG) (Kuin et al, 1994), and by administration of vasoactive drugs like hydralazine (Newell et al, 1992). $^{31}$P Magnetic resonance spectroscopy (MRS) studies have revealed that only the extracellular pH (pHe) is reduced by these interventions (Hwang et al, 1991; Kuin et al, 1994), which restricts their application in vivo to weakly acidic drugs, acid labile prodrugs (Tietze et al, 1989) and camptothecins (Gabr et al, 1997).

Some studies, therefore, have concentrated on reduction of the intracellular pH (pHi) of tumours by inhibition of membrane-based exchangers involved in the regulation of pHi. The two major exchangers are the $Na^+/H^+$ antiport, which can be inhibited by amiloride and its analogues, and the $Na^+$-dependent $HCO_3^-/Cl^-$ exchanger, inhibited by stilbene derivatives such as 4,4′-diisothiocyanostilbene-2,2′-disulphonic acid (DIDS). Furthermore, the ionophore nigericin is able to reduce the pHi by allowing exchange of intracellular $K^+$ for extracellular $H^+$ (Rotin et al, 1987; Newell and Tannock, 1989; Yamagata and Tannock, 1996). Wood et al (1995) have shown that equilibration of the pHi with pre-existing low pHe of a radiation-induced fibrosarcoma (RIF-1) tumour by nigericin can potentiate melphalan-related tumour response, whereas a combination of DIDS with amiloride increased the

*Received 27 January 1998*
*Revised 18 June 1998*
*Accepted 25 June 1998*

*Correspondence to:* LA Smets, The Netherlands Cancer Institute, Department of Experimental Therapy, 120 Plesmanlaan, NL 1066 CX Amsterdam, The Netherlands thermosensitivity of SCK mammary carcinoma (Song et al, 1994). Moreover, because a protracted lowering of the intracellular pH can be cytotoxic by itself (Rotin et al, 1987), Yamagata and Tannock (1996) were able to demonstrate that prolonged administration of nigericin (6 mg $kg^{-1}$) stopped net tumour growth during the infusion time and achieved tumour reduction by nigericin combined with DIDS and the amiloride analogue 5-(*N*-ethyl-*N*-isopropyl) amiloride (EIPA), after lowering tumour pHe from 6.9 to approximately 6.8 by the vasoactive drug hydralazine.

Potentiation of anti-cancer drug activity and the cytotoxicity of low pHi itself are dependent on both the duration and the degree of tumour acidification (Rotin et al, 1987; Yamagata and Tannock, 1996). In an attempt to optimize pHi reduction, MIBG/glucose treatment, which selectively reduces the extracellular pH down to 6.2 for several hours (Kuin et al, 1994), was combined with nigericin or amiloride and DIDS. To investigate the relative contributions of reduced pHe and pHi on the cytotoxicity of several anti-cancer drugs and the possible interference of MIBG with drug action, unrelated to the pH-lowering capacity, we used for practical reasons a L1210 leukaemic cell model. Subsequently, we investigated in RIF-1-bearing mice the optimal conditions of a non-toxic protocol of MIBG/glucose treatment in combination with nigericin or amiloride and DIDS. The analogue benzylguanidine (BG), with comparable antimitochondrial effects, was used as an alternative for MIBG to avoid nephrotoxicity associated with high dose MIBG (Kuin et al, 1998). Finally, we investigated the effect of low pHe and pHi on the in vivo tumour response to the anti-cancer drugs chlorambucil, mitomycin C and melphalan.

### MATERIALS AND METHODS

### Drugs

MIBG was purchased from EMKA Chemie (Markgröningen, Germany). BG was synthesized as described before (Van den Berg et al, 1997). D(+)glucose monohydrate was obtained from Merck (Darmstadt, Germany). 2-Deoxy-D-[1-$^3$H]glucose (specific activity 17.4 Ci $mmol^{-1}$) and [$^{51}$Cr]EDTA (specific activity 1–2 mCi $mg^{-1}$) were purchased from Amersham International (Buckinghamshire, UK). EO9 (3-hydroxymethyl-5-aziridinyl-1-methyl-2-(1H-indole-4,7-dione)prop-β-en-α-ol) was kindly provided by Dr JH Beijnen (Slotervaart Hospital, The Netherlands Cancer Institute, Amsterdam, The Netherlands), cisplatin was obtained from Bristol-Myers Squibb (Weesp, The Netherlands) and all other drugs were from Sigma (St. Louis, MO, USA).

For in vitro experiments, mitomycin C and doxorubicin were dissolved in sodium chloride 0.9% to 1 mg $ml^{-1}$. Chlorambucil was dissolved in DMSO (4 mg $ml^{-1}$) and melphalan, EO9 and nigericin in ethanol (99% v/v) to 0.5, 0.5 and 1 mg $ml^{-1}$ respectively.

In animal experiments, a standard pharmaceutical preparation of melphalan was used (Alkeran; Wellcome Foundation, UK) and injected within 30 min after dissolving. Chlorambucil was dissolved directly before use in acidified ethanol (4.8 ml concentrated hydrochloric acid in 95% v/v ethanol) and diluted 1:9 with propylene glycol/dipotassium hydrogen phosphate buffer (20 g dipotassium phosphate plus 450 ml propylene glycol in a final volume of 1 l), final pH 7.4 (Lee et al, 1986). MIBG and BG were dissolved during 16 h at 37°C in phosphate-buffered saline (PBS). Before use, glucose was added to a concentration of 0.15 or 0.30 g $ml^{-1}$. DIDS and amiloride were dissolved in PBS and demineralized water respectively.

### Cells and experimental conditions

L1210 murine leukaemia cells were routinely cultured in RPMI-1640 medium (Gibco Europe, The Netherlands), supplemented with 10% fetal calf serum (Gibco Europe), 100 U $ml^{-1}$ penicillin, 100 μg $ml^{-1}$ streptomycin and 60 μM β-mercaptoethanol, at 37°C in a humidified atmosphere with 5.6% carbon dioxide. The population doubling time was 12 h.

During MIBG exposure (10 μg $ml^{-1}$) for 4 h, cells were incubated at a density of $5 \times 10^6$ cells $ml^{-1}$ in RPMI-1640 medium without sodium bicarbonate (Gibco Europe). The intracellular pH was measured by using the fluorescent dye 2′,7′-bis(carboxyethyl)-5,6-carboxyfluorescein-triacetoxymethylester (BCECF-AM) as described previously (Atema et al, 1993). The extracellular pH was recorded simultaneously with a silver/silver chloride/gel-filled/sealed Broadley James electrode connected to a Becton Model 32 pH-meter. Glucose and lactate levels in the medium were estimated before and after the incubation with MIBG by routine assays (Boehringer Mannheim, Germany). ATP levels were measured at different incubation periods: samples of $\pm 5 \times 10^6$ cells were extracted with cold perchloric acid (0.5 M). The extracts were neutralized with 2 M potassium hydroxide in 0.3 M 4-morpholino-propane sulphonic acid and stored at –70°C. Samples were diluted in 0.1 M Tris/2 mM EDTA (pH 7.75) and ATP was measured fluorimetrically using luciferin-containing Monitoring Reagent (BioOrbit, Finland). Intracellular ADP levels were converted to ATP by adding phosphoenolpyruvate (3.2 mM) and pyruvate kinase (0.16 mg $ml^{-1}$). The increase in ATP content was used as a measure for the ADP concentration. ATP and ADP levels were correlated to the protein content determined according to the protocol of Bradford (1976).

### Cell survival experiments

For single cell survival experiments, anti-cancer drugs were added at different concentrations to the cell suspension ($5 \times 10^6$ cells $ml^{-1}$) during the last 2 h of the 4-h MIBG incubation period.

To test for the contribution of reduced extra- and intracellular pH, cells ($10^6$ cells $ml^{-1}$) were incubated during 4 h with anti-cancer drugs at different pHe and pHi conditions. To this end, sodium bicarbonate-free RPMI incubation medium was acidified with lactic acid to pH 6.5, and nigericin (10 μM) was added to equilibrate the pHi with the pHe. Incubations at pHe 6.5 without nigericin and at pHe 7.4 with and without nigericin served as controls. The corresponding pHi values were confirmed in parallel cultures.

After exposure, the cell suspensions were diluted with buffered RPMI medium and suspended in a semisolid plating medium consisting of RPMI-1640 supplemented with 20% conditioned medium and 0.6% carboxymethylcellulose (Fluka, Buchs, Switzerland). Cell suspensions of 10 ml, containing 300 or 3000 cells were divided over three wells of a six-well cluster plate (Costar, Cambridge, MA, USA). Macroscopic colonies were counted after 8–9 days and survival was calculated as percentage of controls without anti-cancer drugs. Dose-reduction factors at 50% survival ($DRF_{50}$) were calculated by dividing the $IC_{50}$ at physiological pH by that at low pH.

### Animals and tumour acidification

All animal experiments were carried out in accordance with protocols approved by the experimental animal welfare committee of the

 © Cancer Research Campaign 1999

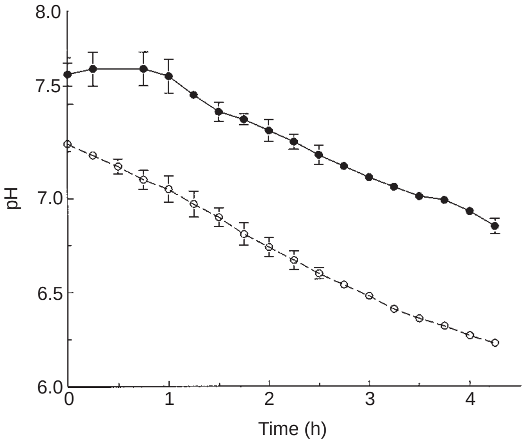

**Figure 1** Extracellular (open symbols) and intracellular pH (closed symbols) of L1210 cells at high cell density (5×10$^6$ cells ml$^{-1}$) exposed to MIBG (10 µg ml$^{-1}$) during 4 h, measured by pH electrode and the fluorescent dye BCECF-AM respectively. Means ± s.d. of three individual experiments. Error bars if not shown are within the size of the symbols institute and conform to national and European regulations for animal experimentation. According to inhouse rules, mice were observed at least twice daily for treatments with unknown toxicity and sacrificed at signs of severe discomfort and weight loss of 15%.

RIF-1 cells from liquid nitrogen stocks were propagated in tissue culture and transplanted at restricted passage numbers according to a previously described protocol (Twentyman et al, 1980). Cells (10$^5$ in 0.1 ml) were s.c. injected on the back of syngeneic male C3H/Km mice weighing 28–35 g at an age of 8–10 weeks. Two weeks after inoculation, tumour diameters reached 3–5 mm and treatment was started.

For tumour-specific acidification, mice were treated by MIBG (30 mg kg$^{-1}$) or BG (37 or 43 mg kg$^{-1}$), in combination with different doses of glucose (1.5 or 3.0 g kg$^{-1}$). Doses were chosen based on absence of reduced renal clearance and proven mitochondrial inhibition in vivo (Kuin et al, 1998). To assay for the effect on tumour metabolism, glucose and lactate levels and deoxyglucose uptake were measured as described previously (Kuin et al, 1994). Briefly, for glucose and lactate measurements, tumours were excised 3 h after treatment, frozen, ground to powder and suspended in demineralized water. Glucose and lactate levels were estimated by routine assays (Boehringer Mannheim) and related to the protein content. 2-Deoxy-D-[1-$^3$H]glucose (10 µCi 100 µl$^{-1}$) was administered i.v. 2 h after treatment. After 1 h, mice were sacrificed and tumour samples of about 50 mg were extracted in 2 ml perchloric acid (0.5 M) during 48 h. Radioactivity of the extracts was assessed by liquid scintillation counting and expressed in percentage of injected dose g$^{-1}$ tumour.

### Tumour treatment protocols

For intracellular acidification, MIBG/glucose and BG/glucose treatments were combined with amiloride and DIDS in doses up to the maximum tolerated dose of 10 and 25 mg kg$^{-1}$ respectively (IF Tannock, personal communication). The drugs were injected at low pHe conditions; 2.5 and respectively 3 h after MIBG/glucose or BG/glucose treatment of which the 30 min interval served to prevent intraperitoneal coprecipitation of the drugs. Melphalan (8 mg kg$^{-1}$) was injected at 3.5 h and mitomycin C, administered in two doses of 2.5 mg kg$^{-1}$, at 3.5 and 6.5 h after start of the treatment. Chlorambucil (10 mg kg$^{-1}$) was injected 3 h after MIBG/glucose treatment without amiloride and DIDS.

Melphalan was injected i.v. and all other drugs i.p. in a volume of 0.01 ml g$^{-1}$ body weight. Control animals received corresponding volumes of PBS. Tumour growth, measured by callipers in three orthogonal diameters, was documented daily, as were body weight and general wellbeing. Tumour response was expressed as the regrowth time to 200% of the pretreatment volume.

### Renal function and melphalan distribution

Renal function was measured by [$^{51}$Cr]EDTA clearance as described previously (Kuin et al, 1998). In short, 4.5 h after MIBG/glucose treatment, mice were injected i.p. with 10 µCi [$^{51}$Cr]EDTA in 100 µl. Thirty minutes after the injection, blood samples were taken from the retro-orbital sinus and mice were sacrificed. Plasma [$^{51}$Cr]EDTA levels were determined by gamma counting and expressed as percentage of injected dose per ml plasma. Increases in residual plasma [$^{51}$Cr]EDTA levels reflect a reduced renal clearance.

The effect of the treatment with MIBG/glucose plus amiloride/DIDS on melphalan pharmacokinetics was investigated by taking blood samples and excising the tumour 15, 60 and 120 min after melphalan injection. Plasma and tumour tissue were frozen until analysis of melphalan concentrations by high-performance liquid chromatography (HPLC) assay (Klaase et al, 1994). Mice were sacrificed immediately after blood sampling.

### Statistical analysis

Significance of differences in mean values was determined by Student's *t*-test. A *P*-value $<0.05$ was judged to be of statistical significance.

### RESULTS

### Antiproliferative and metabolic effects of MIBG on L1210 cells

MIBG at 10 µg ml$^{-1}$ inhibited the mitochondrial respiration of L1210 cells, comparable as described previously (Loesberg et al, 1990), resulting in enhanced glucose consumption (control, 0.32 ± 0.04 µmol 10$^6$ cells$^{-1}$ 4 h$^{-1}$; MIBG, 0.54 ± 0.06 µmol 10$^6$ cells$^{-1}$ 4 h$^{-1}$) and lactate production (control, 0.56 ± 0.08 µmol 10$^6$ cells$^{-1}$ 4 h$^{-1}$; MIBG, 0.94 ± 0.04 µmol 10$^6$ cells$^{-1}$ 4 h$^{-1}$). The pH of the sodium bicarbonate-free incubation medium decreased down to pH 6.2. Addition of recorded amounts of lactate to fresh medium revealed that lactate production alone was responsible for the decrease in pH. The intracellular pH also decreased but a pH gradient of 0.6 pH units was maintained (Figure 1), corresponding to previous studies with acidification by addition of lactic acid (Atema et al, 1993). The pHe of control cells at this high cell density did not decrease below pH 6.8.

The ATP levels remained stable during the incubation period and were equal for untreated (26 ± 3 µmol ATP g$^{-1}$ protein) and MIBG-exposed cells (25 ± 4 µmol ATP g$^{-1}$ protein). ADP levels were below detection levels at both conditions, indicating an unchanged and high energy state after MIBG treatment. The plating efficiency of the L1210 cells (>80%) was not affected by

© Cancer Research Campaign 1999

**Table 1** Dose-reduction factors (DRF) of a 2-h exposure time of several anti-cancer drugs to L1210 cells calculated by the $IC_{50}$ at physiological pH conditions over the $IC_{50}$ after incubation at low pH (down to pH 6.2) due to MIBG exposure. Results represent mean ± s.d. of 3–5 experiments. The relevant pH condition is deduced from results of experiments demonstrated in Figures 2, 3 and 4

| | DRF 50% | pH Condition |
|---|---|---|
| Cisplatin | 2.0 ± 0.4 | pHi |
| Mitomycin C | 1.3 ± 0.3 | pHi |
| EO9 | 6.2 ± 1.5 | pHi |
| Melphalan | 2.8 ± 0.6 | pHi |
| Chlorambucil | 2.2 ± 0.4 | pHe |
| Doxorubucin | 0.64 ± 0.07 | pHe |

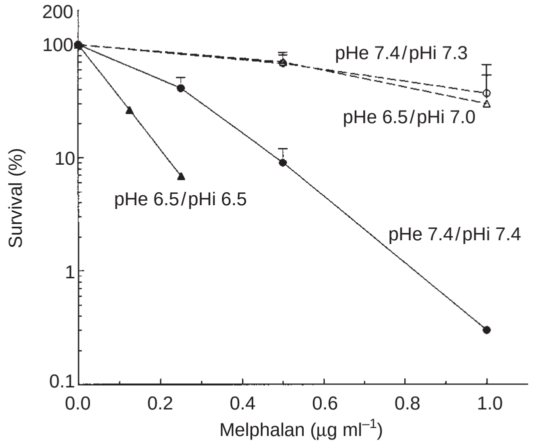

**Figure 2** Survival of L1210 cells ($10^6$ cells $ml^{-1}$) after a 4-h exposure to melphalan at low pHe conditions acidified by lactic acid (triangles) or at physiological pHe (circles), with (closed symbols) or without (open symbols) nigericin to equilibrate pHe with pHi. Means ± s.d. of three individual experiments

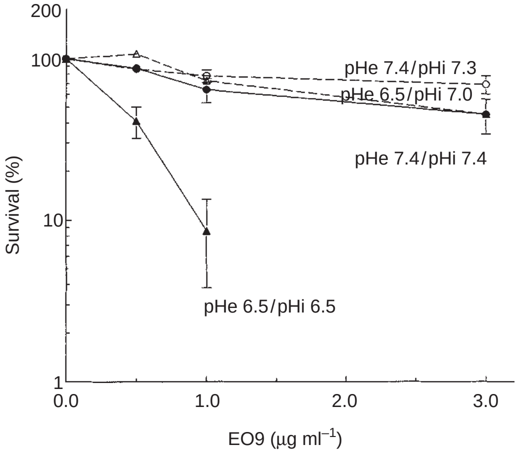

**Figure 3** Survival of L1210 cells ($10^6$ cells $ml^{-1}$) after a 4-h exposure to EO9 at low pHe conditions acidified by lactic acid (triangles) or at physiological pHe (circles), with (closed symbols) or without (open symbols) nigericin to equilibrate pHe with pHi. Means ± s.d. of three individual experiments

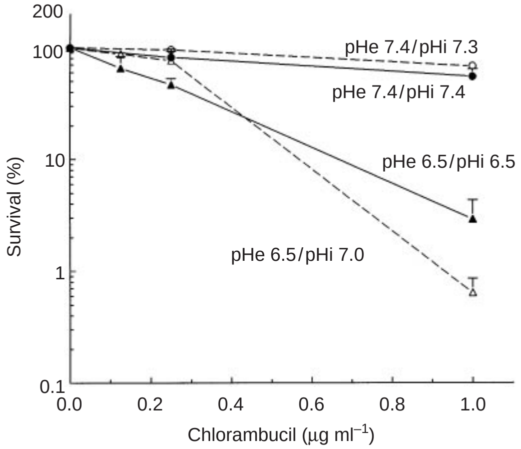

**Figure 4** Survival of L1210 cells ($10^6$ cells $ml^{-1}$) after a 4-h exposure to chlorambucil at low pHe conditions acidified by lactic acid (triangles) or at physiological pHe (circles), with (closed symbols) or without (open symbols) nigericin to equilibrate pHe with pHi. Means ± s.d. of three individual experiments

MIBG-induced decrease in pH under these experimental conditions.

The adherent RIF-1 cells could not be brought to high cell densities or used in convenient FACS-assisted pHi measurements and were therefore not suitable for these in vitro studies.

### Cytotoxicity of anti-cancer drugs to L1210 cells at physiological and low pH

After 2 h preincubation with MIBG, several anti-cancer drugs in increasing concentrations were added for another 2 h. The low pH induced by MIBG protentiated the cytotoxicity of the anti-cancer drugs melphalan, cisplatin, mitomycin C, EO9, and chlorambucil, whereas doxorubicin was less cytotoxic at low pH compared with physiological pH conditions. The level of potentiation of the drugs is expressed in Table 1 as the dose-reduction factor at 50% survival ($DRF_{50}$). Moreover, if MIBG was added at physiological pH to buffered RPMI medium, the pH was not affected and no enhancement was observed with melphalan, EO9 or chlorambucil, excluding a synergistic effect of MIBG with the cytostatic drugs per se.

To specifically investigate the role of low pHi in the observed potentiations, we compared survival after a 4-h incubation period at pHe 7.4 and 6.5, with and without nigericin (1 μM). Addition of lactic acid to reach a pHe of 6.5 was accompanied by a shift of the pHi from 7.3 to 7.0, which further decreased to pH 6.5 within 5 min after addition of nigericin. The latter condition was not toxic to the cells for a 4-h incubation (plating efficiency >70%). However, at pHe values below 6.5, addition of nigericin caused a steep decline in survival (e.g. <0.1% at pHe 6.0) in both L1210 and RIF-1 cells, exactly as reported by other groups (Rotin et al, 1987; Newell and Tannock, 1989). Therefore, to investigate effects of low pHi on the cytotoxicity of anti-cancer drugs, excluding confusing intrinsic toxicity, no pHi conditions below pHi 6.5 were used.

In medium of pH 6.5, melphalan was much more cytotoxic in the presence of nigericin (pHi 6.5) than without nigericin (pHi 7.0) with a $DRF_{50}$ of 11.5 (Figure 2), suggesting that a decrease in pHi of 0.5 units (Figure 1) is mainly responsible for the potentiation observed. However, in control experiments at physiological intra- and extracellular pH, nigericin also potentiated the melphalan-related

**Table 2** Tumour acidification after MIBG or BG treatment combined with glucose, related to tumour lactate levels and [$^3$H]deoxyglucose uptake

| | Tumour lactate levels (mmol g$^{-1}$ protein) | [$^3$H]Deoxyglucose uptake (% uptake g$^{-1}$ tumour) |
|---|---|---|
| PBS | 0.45 ± 0.09[a] | 2.7 ± 1.8 |
| MIBG 40 mg kg$^{-1}$ + glucose 1.5 g kg$^{-1}$[b] | 0.60 ± 0.22[c] | 4.1 ± 0.7[c] |
| MIBG 30 mg kg$^{-1}$ + glucose 1.5 g kg$^{-1}$ | 0.66 ± 0.13[c] | 4.5 ± 2.4[c] |
| BG 43 mg kg$^{-1}$ + glucose 1.5 g kg$^{-1}$ | 0.42 ± 0.05 | 5.4 ± 1.1[c] |
| BG 43 mg kg$^{-1}$ + glucose 3.0 g kg$^{-1}$ | 0.64 ± 0.08[c] | n.d.[d] |

[a]Mean ± s.d. of 6–8 animals; [b]Kuin et al (1994); [c]Different from control, $P < 0.05$; [d]not determined.

**Table 3** Renal function measured by [$^{51}$Cr]EDTA clearance. [$^{51}$Cr]EDTA was injected 4.5 h after MIBG or BG treatment and 30 min later residual plasma levels were measured

| Treatment | | | [$^{51}$Cr]EDTA clearance (% [$^{51}$Cr]EDTA ml$^{-1}$ plasma) |
|---|---|---|---|
| *t* = 0 | *t* = 2.5 h | *t* = 3 h | |
| PBS | | | 1.9 ± 0.5[a] |
| MIBG 40/glucose 1.5[b] | | | 4.0 ± 1.6[c] |
| MIBG 30/glucose 1.5 | | | 2.0 ± 0.2 |
| MIBG 30/glucose 1.5 | Amiloride 5 | DIDS 12.5 | 2.2 ± 2.2 |
| MIBG 30/glucose 1.5 | Amiloride 10 | DIDS 25 | 4.4 ± 0.7[c] |
| BG 43/glucose 1.5 | Amiloride 10 | DIDS 25 | 2.2 ± 0.5 |
| BG 37/glucose 3.0 | Amiloride 10 | DIDS 25 | 3.3 ± 0.5[c] |
| BG 37/glucose 3.0 | | | 3.2 ± 0.4[c] |
| Glucose 3.0 | | | 3.4 ± 0.3[c] |

[a]Mean ± s.d. of six animals; [b]Kuin et al (1998); [c]Different from control, $P < 0.05$.

**Table 4** Tumour response to the interventions alone and in combination with melphalan expressed as the tumour regrowth time to 200% of day 0

| | Regrowth time (days) mean ± s.d. ($n$ = 6–8) |
|---|---|
| Control | 1.5 ± 0.7 |
| MIBG/glucose + amiloride DIDS[a] | 2.2 ± 0.4[c] |
| BG/glucose + amiloride DIDS[b] | 2.5 ± 0.3[c] |
| Melphalan | 5.7 ± 0.6 |
| MIBG/glucose + melphalan | 6.8 ± 0.9[c] |
| BG/glucose + melphalan | 6.9 ± 1.7 |
| Amiloride/DIDS + melphalan | 6.7 ± 0.7[d] |
| MIBG/glucose + amiloride/DIDS + melphalan | 7.4 ± 0.8[d] |
| BG/glucose + amiloride/DIDS + melphalan | 8.1 ± 0.7[d] |

[a]5 mg kg$^{-1}$ amiloride/12.5 mg kg$^{-1}$ DIDS; [b]10 mg kg$^{-1}$ amiloride/25 mg kg$^{-1}$ DIDS; [c]Different from control, $P$ <0.05; [d]Different from melphalan alone, $P < 0.05$.

cell kill with a $DRF_{50}$ of 3.8. This unexpected, pH-independent synergism was specific for the combination of melphalan and nigericin and not found for cisplatin, mitomycin C, E09, chlorambucil or doxorubicin. Moreover, amiloride and DIDS in non-toxic concentrations of 0.2 mM and 1 mM, respectively, did not enhance melphalan toxicity at pH 7.4. Attempts to directly test the pHi effect on melphalan cytotoxicity by replacing nigericin with amiloride and DIDS were inconclusive because the time to equilibrate pHi with pHe by these drugs took up to 3 h, and prolonged exposure to amiloride and DIDS at pH 6.5 was toxic on its own.

As for melphalan, cytotoxicity of E09 was not enhanced at pHe 6.5 (Figure 3), but strongly potentiated after simultaneous reduction of the pHi to 6.5. In comparable experiments with mitomycin C and cisplatin, the potentiations were also mainly due to a reduced pHi, as summarized in Table 1. In contrast, the cytotoxicity of chlorambucil was strongly potentiated at pHe 6.5 (Figure 4), but not further enhanced by simultaneous pHi reduction by nigericin. Similar experiments with doxorubicin confirmed the reported inhibition of its cytotoxicity at low pHe conditions (Alabaster et al, 1989). From this screen, chlorambucil would appear the most interesting drug to combine in animal studies with MIBG/glucose treatment alone, whereas potentiation of melphalan, mitomycin C, EO9 and cisplatin requires the inclusion of pH-equilibrating drugs in the protocols.

© Cancer Research Campaign 1999 

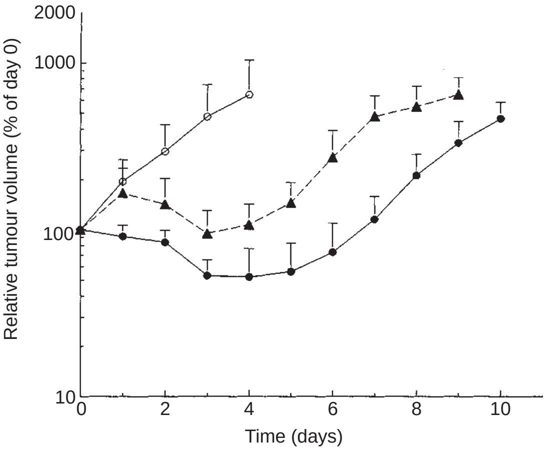

**Figure 5** Relative tumour growth of RIF-1 tumour in C3H/Km mice after treatment with PBS (open circle), melphalan (8 mg $kg^{-1}$ i.v., closed triangle) or BG/glucose plus amiloride/DIDS and melphalan (closed circle). Means ± s.d. ($n$ = 6)

**Table 5** Time-schedule dependency of the combined treatment on the tumour response

| | Time of treatment | | Regrowth time (days) mean ± s.d. ($n$ = 6–8) |
|---|---|---|---|
| **BG/glucose** | **Amiloride/DIDS** | **Melphalan** | |
| 0 h | 0–0.5 h | 3.5 h | 8.4 ± 1.2 |
| [a]0 h | 2.5–3 h | 3.5 h | 8.1 ± 0.7 |
| 0 h | 2.5–3 h | 6 h | 8.2 ± 1.3 |

[a]Standard protocol.

### Tumour acidification

To simultaneously reduce extra- and intracellular tumour pH, we combined the intervention MIBG/glucose with pH-equilibrating drugs nigericin or amiloride and DIDS. In previous studies, we have demonstrated that 40 mg $kg^{-1}$ MIBG plus 1.5 g $kg^{-1}$ glucose reduces tumour pH for several hours down to pH 6.2 (Kuin et al, 1994) without affecting tumour growth (Gabr et al, 1997). Inclusion of 10 mg $kg^{-1}$ amiloride and 25 mg $kg^{-1}$ DIDS to this MIBG/glucose protocol induced a significant anti-tumour response, i.e. a reduction of tumour volume after 2 days to 59% ± 27% of the pretreatment value with a corresponding increase in tumour regrowth time from 1.5 to approximately 4.3 days. Similar results were obtained with nigericin (1.5 mg $kg^{-1}$) if administered between 1 and 6 h after MIBG/glucose treatment (A Gabr, personal communication). These experiments indicate that nigericin and amiloride plus DIDS were able to reduce the intracellular pH below a critical level for cell kill at an interstitial pH of 6.2. After MIBG at 40 mg $kg^{-1}$ in combination with amiloride and DIDS, body weights progressively decreased and animals were sacrificed when weight loss exceeded 15%, mainly after resumption of tumour growth. The combination of MIBG with nigericin did not significantly affect body weights but caused acute lethality in one out of five mice. Moreover, MIBG itself induces, at 40 mg $kg^{-1}$, reversible reduction in renal function (Kuin et al, 1998). A series of experiments was, therefore, performed to establish conditions of tumour acidification with minimal toxicity. In addition to lower doses of MIBG, we also tested the MIBG analogue BG, which mimics the antimitochondrial effects of MIBG but is less nephrotoxic (Van den Berg et al, 1997; Kuin et al, 1998). Tumour pH response was evaluated biochemically by increases in tumour lactate levels and [$^3$H]deoxyglucose uptake in comparison to the previously described effects of MIBG/glucose treatment (Table 2). BG at 43 mg $kg^{-1}$ was only effective in increasing tumour lactate levels when combined with 3.0 g $kg^{-1}$ glucose. This higher dose of glucose did neither change tumour lactate levels when given alone nor further enhance tumour lactate levels when combined with MIBG (data not shown). Addition of i.v. injected melphalan to BG/glucose resulted in acute effects, sometimes even lethal. Therefore, the BG dose was decreased to 37 mg $kg^{-1}$, i.e. twice the molar dose of 30 mg $kg^{-1}$ MIBG, which did not demonstrate these acute effects.

Table 3 summarizes the effects of the various treatments including amiloride and DIDS on renal function measured by [$^{51}$Cr]EDTA clearance. High doses of glucose (3 g $kg^{-1}$) affect the [$^{51}$Cr]EDTA kinetics independent of renal toxicity as described previously (Kuin et al, 1998). The ionophore nigericin (1.5 mg $kg^{-1}$) slightly reduced renal function when given alone (from 1.8% ± 0.3% to 2.7% ± 0.8% [$^{51}$Cr]EDTA $ml^{-1}$ plasma; $P$ = 0.03) or in combination with 37 mg $kg^{-1}$ BG plus 3.0 g $kg^{-1}$ glucose (3.7% ± 2.2% [$^{51}$Cr]EDTA $ml^{-1}$ plasma; $^P$ = 0.09), but strongly affected renal function when combined with 30 mg $kg^{-1}$ MIBG plus 1.5 g $kg^{-1}$ glucose (6.9% ± 1.6% [$^{51}$Cr]EDTA $ml^{-1}$ plasma; $P$ < 0.001).

The finally chosen non-toxic interventions were 30 mg $kg^{-1}$ MIBG plus 1.5 g $kg^{-1}$ glucose followed by 5 mg $kg^{-1}$ amiloride ($t$ = 2.5 h) and 12.5 mg $kg^{-1}$ DIDS ($t$ = 3 h), and 37 mg $kg^{-1}$ BG plus 3.0 g $kg^{-1}$ glucose followed by 10 mg $kg^{-1}$ amiloride and 25 mg $kg^{-1}$ DIDS at similar intervals. These will be referred to in further experiments as MIBG/glucose plus amiloride/DIDS and BG/glucose plus amiloride/DIDS respectively.

### Tumour response to anti-cancer drugs after reduction of extra- and intracellular pH

The two interventions described above each induced a slight but significant delay in tumour regrowth time (Table 4), but had no effect without amiloride and DIDS (data not shown).

Because chlorambucil was in vitro specifically potentiated at a low extracellular pH (Figure 4), this drug was tested after MIBG/glucose treatment without amiloride and DIDS. Chlorambucil (10 mg $kg^{-1}$) caused an increase in tumour growth delay from 1.5 ± 0.8 to 5.3 ± 0.6 days ($P$ < 0.001), but in contrast to the in vitro results the tumour response was not enhanced by a preceding MIBG/glucose treatment (tumour regrowth time 5.8 ± 0.9 days; $P$ = 0.15).

Under standard conditions, the response to mitomycin C (tumour regrowth time 3.8 ± 1.0 days) was enhanced neither by MIBG/glucose or BG/glucose alone nor in combination with amiloride and DIDS. However, a significantly enhanced response by MIBG/glucose plus amiloride/DIDS was observed in a pilot experiment with large tumours of >5 mm diameter, increasing the tumour regrowth time from 3.8 ± 0.5 for mitomycin C alone to 5.2 ± 0.5 days for the combined treatment ($n$ = 6; $P$ < 0.001).

The mitomycin C derivative EO9 demonstrated severe toxicity at doses above 5 mg $kg^{-1}$ that included peritoneal oedema, necrosis of the intestines and, in some cases, acute peritonitis, liver toxicity and hyperaemic bone marrow. Because the maximum tolerated dose of 5 mg $kg^{-1}$ did not result in a tumour response, studies with EO9 were discontinued.

*© Cancer Research Campaign 1999*

Melphalan (8 mg kg$^{-1}$; i.v.) was slightly potentiated by MIBG/glucose, BG/glucose or amiloride/DIDS (Table 4). The combined treatments further enhanced the tumour response (Table 4), and the additional tumour growth delay of 2.4 days for BG/glucose plus amiloride/DIDS is illustrated in Figure 5. Because of the delayed pH-equilibrating effects of amiloride and DIDS observed in vitro, simultaneous administration of BG/glucose and amiloride/DIDS or delayed administration of melphalan were compared with the standard protocol. All schedules appeared to be equally effective (Table 5).

### Pharmacokinetics and toxicity

The treatments MIBG/glucose plus amiloride/DIDS and BG/glucose plus amiloride/DIDS combined with melphalan, mitomycin C or chlorambucil did not decrease body weight by more than 10%, and no visible signs of illness were observed in any of the combinations. To confirm that melphalan pharmacokinetics were not changed in accordance with the absence of reduced [$^{51}$Cr]EDTA clearance, melphalan plasma levels were measured. At 15, 60 and 120 min after drug administration, the melphalan plasma levels were 3.7 ± 0.7, 1.0 ± 0.1 and 0.33 ± 0.05 μg ml$^{-1}$ respectively. The tumour melphalan levels reflected plasma concentrations. MIBG/glucose or BG/glucose treatment with or without the addition of amiloride and DIDS did not significantly affect melphalan levels in plasma and tumours.

### DISCUSSION

Although tumour-selective pHe reduction is of demonstrated importance in the response to the topoisomerase I inhibitor camptothecin (Gabr et al, 1997), pH-mediated potentiation of several clinically relevant drugs requires reduction of the intracellular pH (Jähde et al, 1989; Atema et al, 1993). For this purpose, the ionophore nigericin (Wood et al, 1995) and inhibitors of $Na^+/H^+$ and the $Na^+$-dependent $HCO_3^-/Cl^-$ exchanger (Yamagata and Tannock, 1996) have demonstrated efficacy in vivo. Because the level of the intracellular acidification is limited by extracellular pH values (Wood et al, 1995), in the present study we tested the feasibility of selectively reducing the extracellular pH of the tumour before the administration of pH-equilibrating drugs.

The mitochondrial inhibitor MIBG, coadministered with glucose, selectively reduces the intratumoral pH of RIF-1 tumours down to pH 6.2, as demonstrated in a previous study (Kuin et al, 1994). In vitro acidification of murine leukaemic L1210 cells by exposure to MIBG revealed comparable conditions as observed in vivo: enhanced glycolytic flux, no change in the high energy state and pH reduction of mainly the extracellular compartment. Using MIBG for acidification, in vitro potentiation of cisplatin, melphalan, mitomycin C and chlorambucil were comparable with previous studies using hydrochloric acid or lactic acid (Jähde et al, 1989; Atema et al, 1993), and were independent of MIBG itself. In addition, the mitomycin C derivative EO9 was strongly potentiated at low pH conditions. The in vitro studies also confirmed the predominant role of low pHi in these potentiations by stimulated DNA alkylation of cisplatin and melphalan (Jähde et al, 1989; Atema et al, 1993) and enhanced bioreductive activation of mitomycin C and EO9 (Gibson et al, 1992; Philips et al, 1992; Begleiter and Leith, 1993). Furthermore, we confirmed that chlorambucil is exclusively potentiated at low extracellular pH conditions which, as a weak acid with a $pK_a$ value of 5.8, enhances its uptake (Mikkelsen et al, 1985; Skarsgard et al, 1992). The opposite was true for doxorubicin cytotoxicity because of a decreased uptake at low pHe conditions (Alabaster et al, 1989).

Whereas potentiation of melphalan at low pH in the in vitro studies is in agreement with literature, the observed synergism between nigericin and melphalan at physiological pH (Figure 2) was remarkable and basically not understood. Potentiation of DNA alkylation by nigericin is unlikely because no interaction with the structurally related chlorambucil was found. We, therefore, speculate that nigericin can stimulate melphalan uptake by the $Na^+$/amino acid symport. Because enhancement of melphalan activity by nigericin was reported by Wood et al (1995) in only one out of three tumour types, the observed synergism might be restricted to certain cell types, which include L1210 cells.

Nigericin was abandoned early from in vivo studies because of its possible interaction with melphalan, its effect on renal function especially in combination with MIBG, and of its non-specific action and unpredictable side-effects in human patients. The final in vivo interventions of MIBG/glucose or BG/glucose with amiloride and DIDS were not toxic when either applied alone or combined with cytostatic drugs. Moreover, they did not reduce renal clearance which could affect the tumour response by altered pharmacokinetics.

In comparison to our previous study (Kuin et al, 1994), the MIBG dose was reduced from 40 to 30 mg kg$^{-1}$ or substituted for the analogue BG. Comparable increases in tumour lactate levels and deoxyglucose uptake indicated a similar decrease in pHe down to 6.2, as was measured previously by pH electrodes. Because BG lacks the ability of MIBG to increase plasma glucose levels by sympathomimetic, stress-related gluconeogenesis (Kuin et al, 1994), more glucose (3 g kg$^{-1}$) was needed for adequate support of glycolysis. Although $^{31}$P MRS was not available to measure the pHi, the pH modulation in the presence of amiloride and DIDS revealed slight but significant anti-tumour responses, indicating a reduction of the intracellular pH ≤ 6.5 for at least certain tumour areas and sufficient time to induce cell kill (Rotin et al, 1987; Newell and Tannock, 1989). This level of acidification is in reasonable agreement with a calculated pHi of about 6.6, assuming full equilibration of protons from the extracellular volume of 30% of RIF-1 tumours (Evelhoch et al, 1984) at pHe 6.2. Variation in the schedule of the combined treatment with melphalan (Table 5) indicated that an effective low intratumoral pH persisted for at least 6 h after the start of the treatment.

In contrast to the in vitro results, low tumour pHe did not enhance chlorambucil-related tumour response. This was also in conflict with reports on enhanced cell kill and tumour response to chlorambucil simultaneously given with the vasoactive drugs hydralazine (Skarsgard et al, 1992) or flavone acetic acid (Parkins et al, 1994) to manipulate the pH. Perhaps the effect of drug trapping by a strongly reduced tumour blood flow is more important than a reduced pHe in these responses.

Reduced pHe combined with amiloride and DIDS enhanced mitomycin C tumour response only in large sized tumours (>5 mm diameter). Apparently, the manifestation of pH-mediated potentiation of mitomycin C is dependent on conditions that specifically exist in larger tumours, e.g. the extent of hypoxia and lower perfusion rate (Vaupel et al, 1989). Moreover, Nishiyama et al (1997) have demonstrated that tumour pH manipulation by MIBG/glucose increased mitomycin C activity in some but not all tumour types. Although the experimental bioreductive agent EO9 was most promising in vitro (Table 1), its low therapeutic index

© Cancer Research Campaign 1999

observed in the present experiments agrees with observations during clinical studies (Dirix et al, 1996).

So far, the combined treatment was most effective on melphalan tumour response, which is in agreement with in vitro findings. Amiloride/DIDS alone enhanced melphalan-induced tumour growth delay by 1 day and also pHe reduction by MIBG/glucose or BG/glucose alone slightly enhanced melphalan tumour response. Accordingly, the tumour growth delay of the combined treatment of 2.4 days might be due to a combined pH reduction. The effects are less than the tumour growth delay of about 5 days reported for the same tumour treated with melphalan plus nigericin (Wood et al, 1995). The difference might be due to slower intracellular acidification by amiloride and DIDS and a possible contribution of synergism between nigericin and melphalan. Moreover, it is of note that in these studies melphalan alone caused minimal tumour growth delay compared with 4 days in the present study.

In conclusion, we have developed a non-toxic intervention using the mitochondrial inhibitor MIBG, or its analogue BG, plus glucose in combination with amiloride and DIDS for a sustained and selective reduction of tumour pHe to approximately 6.2 and pHi to about 6.5–6.6. In contrast to nigericin, glucose and amiloride are established clinical drugs and MIBG can safely be given intravenously in doses up to 135 mg $m^{-2}$ (B Taal, Netherlands Cancer Institute, personal communication). An important limitation in the present and a previous study (Gabr et al, 1997), however, is that bolus injection of cytostatic drugs with short half-lives are suboptimal to fully exploit the protracted pH manipulation achieved by our protocol. The use of osmotic minipumps in future studies might obviate this limitation.

Although intracellular tumour acidification per se is a potential cytostatic intervention (Newell and Tannock, 1989; Yamagata and Tannock, 1996), it contributed minimally to the potentiations observed in the present study. Reduction of the intracellular pH in vivo below critical values is thought to be self-limiting by inhibition of glycolytic key enzymes (Mansour, 1972) and a restricted amount of protons in the extracellular volume. Accordingly, reduction of the intracellular pH much below 6.5 would not seem an attractive goal. Our results and those of Yamagata and Tannock (1996) indicate that the anti-tumour effects will be maximal with prolonged exposure to both pH-equilibrating drugs and selected pHi-sensitive anti-cancer drugs.

### ACKNOWLEDGEMENTS

The authors gratefully acknowledge Dr J van den Berg (Division of Experimental Therapy, The Netherlands Cancer Institute) for the synthesis of BG, C van Beers and R Lodewijks (Department of Clinical Chemistry, The Netherlands Cancer Institute) for glucose and lactate determinations, E Noteboom (Division of Cell Biology, The Netherlands Cancer Institute) for FACS-assisted pHi measurements and R van Gijn (Department of Pharmacy and Pharmacology, Slotervaart Hospital, The Netherlands Cancer Institute) for melphalan plasma level determinations. This work was supported by the Dutch Cancer Foundation, Grant NKI 91-14.

### REFERENCES

Alabaster O, Woods T, Ortiz-Sanchez V and Jahangeer S (1989) Influence of microenvironmental pH on adriamycin resistance. *Cancer Res* **49**: 5638–5643

Atema A, Buurman KJH, Noteboom E and Smets LA (1993) Potentiation of DNA-adduct formation and cytotoxicity of platinum-containing drugs by low pH. *Int J Cancer* **54**: 166–172

Begleiter A and Leith MK (1993) Role of NAD(P)H:(quinone acceptor) oxidoreductase (DT-diaphorase) in activation of mitomycin C under acidic conditions. *Mol Pharmacol* **44**: 210–215

Bradford MM (1976) A rapid and sensitive method for quantitation of microgram quantities of protein utilizing the principle of protein-dye binding. *Anal Biochem* **72**: 248–254

Dirix LY, Tonnesen F, Cassidy J, Epelbaum R, ten Bokkel Huinink WW, Pavlidis N, Sorio R, Gamucci T, Wolff I, te Velde A, Lan J and Verwey J (1996) EO9 phase II study in advanced breast, gastric, pancreatic and colorectal carcinoma by the EORTC early clinical studies group. *Eur J Cancer* **32**: 2019–2022

Evelhoch JL, Sapareto SA, Jick DEL and Ackerman JJH (1984) In vivo metabolic effects of hyperglycaemia in murine radiation-induced fibrosarcoma: a $^{31}P$ NMR investigation. *Proc Natl Acad Sci USA* **81**: 6496–6500

Gabr A, Kuin A, Aalders M, El-Gawly H and Smets LA (1997) Cellular pharmacokinetics and cytotoxicity of camptothecin and topotecan at normal and acidic pH. *Cancer Res* **57**: 4811–4816

Gibson NW, Siegel D and Ross D (1992) Mitomycin C. *Cancer Chemother Biol Response Modif* **13**: 59–68

Hwang YC, Kim S-G, Evelhoch JL, Seyedsadr M and Ackerman JJH (1991) Modulation of murine radiation-induced fibrosarcoma-1 tumor metabolism and blood flow in situ via glucose and mannitol administration monitored by $^{31}P$ and $^{2}H$ nuclear magnetic resonance spectroscopy. *Cancer Res* **51**: 3108–3118

Jähde E and Rajewsky MF (1982) Tumor-selective modification of cellular microenvironment in vivo: effect of glucose infusion on the pH in normal and malignant rat tissues. *Cancer Res* **42**: 1505–1512

Jähde E, Glüsenkamp K-H, Klünder I, Hülser DF, Tietze L-F and Rajewsky MF (1989) Hydrogen ion-mediated enhancement of cytotoxicity of bis-chloroethylating drugs in rat mammary carcinoma cells in vitro. *Cancer Res* **49**: 2965–2972

Klaase JM, Kroon BBR, Beijnen JH, van Slooten GW and van Dongen JA (1994) Melphalan tissue concentrations in patients treated with regional isolated perfusion for melanoma of the lower limb. *Br J Cancer* **70**: 151–153

Kuin A, Smets L, Volk T, Paans A, Adams G, Atema A, Jähde E, Maas A, Rajewsky MF, Visser G and Wood P (1994) Reduction of intratumoral pH by the mitochondrial inhibitor *m*-iodobenzylguanidine and moderate hyperglycaemia. *Cancer Res* **54**: 3785–3792

Kuin A, Aalders M, van der Valk MA, Frey A, Schmidt HHHW and Smets LA (1998) Renal toxicity of the neuron-blocking and mitochondriotropic agent *m*-iodobenzylguanidine (MIBG). *Cancer Chemother Pharmacol* **42**: 37–45

Lee FYF, Coe P and Workman P (1986) Pharmacokinetic basis for the comparative antitumor activity and toxicity of chlormabucil, phenylacetic acid mustard and β,β-difluorochlorambucil (CB 7103) in mice. *Cancer Chemother Pharmacol* **17**: 21–29

Loesberg C, van Rooij H, Nooijen WJ, Meijer AJ and Smets LA (1990) Impaired mitochondrial respiration and stimulated glycolysis by *m*-iodobenzylguanidine (MIBG). *Int J Cancer* **46**: 276–281

Mansour TE (1972) Phosphofructokinase. *Curr Top Cell Regul* **5**: 1–46

Mikkelsen RB, Asher C and Hicks T (1985) Extracellular pH, transmembrane distribution and cytotoxicity of chlorambucil. *Biochem Pharmacol* **34**: 2531–2534

Newell KJ and Tannock IF (1989) Reduction of intracellular pH as a possible mechanism for killing cells in acidic regions of solid tumors: effects of carbonylcyanide-3-chlorophenylhydrazone. *Cancer Res* **49**: 4477–4482

Newell K, Wood P, Stratford I and Tannock I (1992) Effects of agents which inhibit the regulation of intracellular pH on murine solid tumours. *Br J Cancer* **66**: 311–317

Nishiyama M, Suzuki K, Kumazaki T, Yamamoto W, Toge T, Okamura T and Kurisu K (1997) Molecular targeting of mitomycin C chemotherapy. *Int J Cancer* **72**: 649–656

Parkins CS, Chadwick JA and Chaplin DJ (1994) Enhancement of chlorambucil cytotoxicity by combination with flavone acetic acid in a murine tumour. *Anticancer Res* **14**: 1603–1608

Philips RM, Hulbert PB, Bibby MC, Sleigh NR and Double JA (1992) In vitro activity of the novel indoloquinone EO-9 and the influence of pH on cytotoxicity. *Br J Cancer* **65**: 359–364

Rotin D, Wan P, Grinstein S and Tannock I (1987) Cytotoxicity of compounds that interfere with the regulation of intracellular pH: a potential new class of anticancer drugs. *Cancer Res* **47**: 1497–1504

Skarsgard LD, Chaplin DJ, Wilson DJ, Skwarchuk MW, Vinczan A and Kristl J (1992) The effect of hypoxia and low pH on the cytotoxicity of chlorambucil. *J Radiat Oncol Biol Phys* **22**: 737–741

Song CW, Kim GE, Lyons JC, Makepeace CM, Griffin RJ, Rao GH and Cragoe EJ (1994) Thermosensitization by increasing intracellular acidity with amiloride and its analogues. *Int J Radiat Oncol Biol Phys* **30**: 1161–1169

© Cancer Research Campaign 1999

Thislethwaite AJ, Alexander GA, Moylan III DJ and Leeper DB (1987) Modification of human tumor pH by elevation of blood glucose. *Int J Radiat Oncol Biol Phys* **13**: 603–610

Tietze LF, Neumann M, Möllers T, Fischer R, Glüsenkamp K-H, Rajewsky MF and Jähde E (1989) Proton-mediated liberation of aldophosphamide from a nontoxic prodrug: a strategy for tumor-selective activation of cytocical drugs. *Cancer Res* **49**: 4179–4184

Twentyman PR, Brown JM, Gray JW, Franko AJ, Scoles MA and Kallman RF (1980) A new mouse tumor model system (RIF-1) for comparison of end-point studies. *J Natl Cancer Inst* **64**: 595–604

Van den Berg JD, Smets LA, Rutgers M, Grummels A, Fokkens R, Jonkergauw P and van Rooij H (1997) Chemical characterization and comparative cellular effects of *meta*-iodobenzyl guanidine and benzyl guanidine. *Cancer Chemother Pharmacol* **40**: 131–137

Vaupel P, Kallinowski F and Okunieff P (1989) Blood flow, oxygen and nutrient supply, and metabolic microenvironment of human tumors: a review. *Cancer Res* **49**: 6449–6465

Volk T, Jähde E, Fortmeyer HP, Glüsenkamp K-H and Rajewsky MF (1993) pH in human tumour xenografts: effect of intravenous administration of glucose. *Br J Cancer* **68**: 492–500

Wike-Hooley JL, Haveman J and Reinhold HS (1984) The relevance of tumour pH to the treatment of malignant disease. *Radiother Oncol* **2**: 343–366

Wood PJ, Sansom JM, Newell K, Tannock IF and Stratford IJ (1995) Reduction of tumour intracellular pH and enhancement of melphalan cytotoxicity by the ionophore nigericin. *Int J Cancer* **60**: 264–268

Yamagata M and Tannock IF (1996) The chronic administration of drugs that inhibit the regulation of intracellular pH: in vitro and anti-tumour effects. *Br J Cancer* **73**: 1328–1334

*© Cancer Research Campaign 1999*
